# Conjugation with polyamines enhances the antibacterial and anticancer activity of chloramphenicol

**DOI:** 10.1093/nar/gku539

**Published:** 2014-06-26

**Authors:** Ourania N. Kostopoulou, Ekaterini C. Kouvela, George E. Magoulas, Thomas Garnelis, Ioannis Panagoulias, Maria Rodi, Georgios Papadopoulos, Athanasia Mouzaki, George P. Dinos, Dionissios Papaioannou, Dimitrios L. Kalpaxis

**Affiliations:** 1 Department of Biochemistry, School of Medicine, University of Patras, GR-26504 Patras, Greece; 2 Laboratory of Synthetic Organic Chemistry, Department of Chemistry, University of Patras, GR-26504 Patras, Greece; 3 Division of Hematology, Department of Internal Medicine, School of Medicine, University of Patras, GR-26504 Patras, Greece; 4 Department of Biochemistry and Biotechnology, University of Thessaly, Ploutonos 26, GR-41221 Larissa, Greece

## Abstract

Chloramphenicol (CAM) is a broad-spectrum antibiotic, limited to occasional only use in developed countries because of its potential toxicity. To explore the influence of polyamines on the uptake and activity of CAM into cells, a series of polyamine–CAM conjugates were synthesized. Both polyamine architecture and the position of CAM-scaffold substitution were crucial in augmenting the antibacterial and anticancer potency of the synthesized conjugates. Compounds 4 and 5, prepared by replacement of dichloro-acetyl group of CAM with succinic acid attached to N4 and N1 positions of *N*^8^,*N*^8^-dibenzylspermidine, respectively, exhibited higher activity than CAM in inhibiting the puromycin reaction in a bacterial cell-free system. Kinetic and footprinting analysis revealed that whereas the CAM-scaffold preserved its role in competing with the binding of aminoacyl-tRNA 3′-terminus to ribosomal A-site, the polyamine-tail could interfere with the rotatory motion of aminoacyl-tRNA 3′-terminus toward the P-site. Compared to CAM, compounds 4 and 5 exhibited comparable or improved antibacterial activity, particularly against CAM-resistant strains. Compound 4 also possessed enhanced toxicity against human cancer cells, and lower toxicity against healthy human cells. Thus, the designed conjugates proved to be suitable tools in investigating the ribosomal catalytic center plasticity and some of them exhibited greater efficacy than CAM itself.

## INTRODUCTION

The ribosome is an extremely complex cellular organelle that provides the platform upon which the codons of the mRNA are decoded by aminoacyl-tRNAs. During the successive stages of protein synthesis, the ribosome can interact with a diverse set of additional ligands, like translation factors and antibiotics, which coordinate the function and structure of different regions of the translational machinery to assume the appropriate conformational states which ensure the prospective response.

X-ray crystallography proved to be instrumental in interpreting a wealth set of biochemical data regarding the function of ribosomes fixed in different states and complexed with various classes of ligands (1–3). However, crystallographic analysis provides only a snapshot of ribosome structure and cannot describe in detail the course of conformational changes and interactions by which a ligand is gaining access to the ribosome, nor can it clearly add to our understanding of how signals are transmitted through allosteric networks of the ribosome. Nevertheless, other approaches have been used to dissect more efficiently the dynamic character of the translation process and the plasticity of ribosomal structure, such as kinetics (4), time-resolved footprinting analysis (5), cryo-electron microscopy (6), NMR analysis (7), FRET-based approaches (8), molecular dynamics modeling (9) and biochemical techniques combined with molecular genetics (10). In the present study, we re-examined the dynamic behavior of the ribosome, using a series of polyamine (PA)–chloramphenicol (CAM) conjugates to probe the peptidyl transferase (PTase) region plasticity, and applying kinetic analysis combined with time-resolved footprinting analysis to map the interactions between rRNA and these novel agents.

CAM is a broad spectrum antibiotic, which inhibits protein synthesis by binding to the PTase region of the large ribosomal subunit of bacteria via a two-step mechanism, behaving as a slow binding inhibitor (4,11) and blocking essential ribosomal functions (11–14). Thirteen point mutations or modifications at 11 nucleotides in the central loop of domain V of 23S ribosomal RNA (rRNA) have been identified in bacteria, archaea and mitochondria to be related with decreased sensitivity or resistance against CAM (15–17). Most of these nucleotides change their reactivity against chemical probes upon binding of CAM to the ribosome (4,18,19). Taking into account the molecular size of CAM, such a complicated pattern can be interpreted either by the existence of more than one binding sites of CAM in the ribosome or by conformational changes in ribosomal residues triggered by the bound drug and transmitted over long distances within the PTase region. Therefore, CAM alone or in conjugation with other molecules may be used as an efficient agent for probing the plasticity of the PTase center.

The clinical use of CAM is limited in developed countries, due to its adverse effects that include bone marrow depression and aplastic anemia (20). For this reason, CAM has been modified using a variety of synthetic approaches to acquire an optimized pharmaceutically profile (4,21,22). This fact motivated us to design and synthesize a series of PA–CAM conjugates. We envisaged PA moiety offering additional binding sites to the construct, through its amino functions. In fact, there is cumulative evidence that PAs may be implicated in the binding of CAM to ribosomes (11,23). On the other hand, spermine and spermidine have been found to increase the CAM susceptibility in *Escherichia coli* and other Gram-negative bacteria (24), an effect attributed to the ability of polycations to perturb the outer membrane by displacement of divalent cations existing between adjacent lipo-polysaccharides, and to their potency to inhibit the efflux pumps (25).

Idiosyncratic structural differences between bacterial and eukaryotic ribosomes provide the basis of antibiotic specificity. It should be noted that a very dangerous side-effect of some antibiotics is caused by their ability to diffuse inside mitochondria and inhibit mitochondrial protein synthesis. This happens because mitochondrial ribosomes may be of bacterial origin and share similar structure and, therefore, can be targeted by many antibiotics (26). On the other hand, conjunction with PAs may result in agents capable of selectively exploiting the highly active PA-transporters (PAT) in cancer cells (27). In addition, the PA backbone would recognize the ionic surface of mitochondria and penetrate these organelles (28). Both properties render PA–CAM conjugates promising anticancer agents.

**Figure 1.**
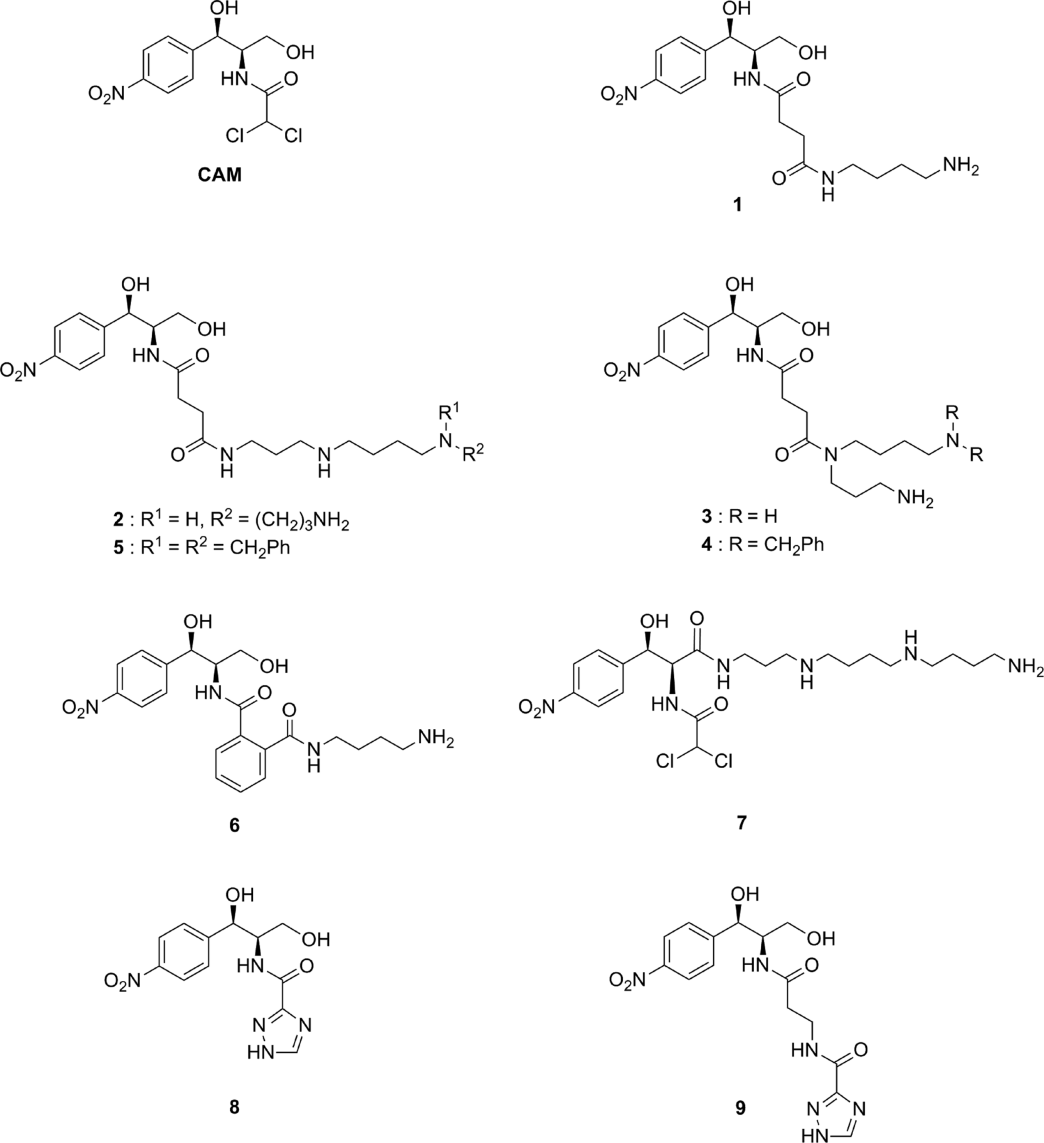
Structures of compounds described in the present work.

Modulating the affinity and selectivity of the PA moiety is another challenge in designing PA–CAM conjugates. We synthesized a series of PA–CAM conjugates (compounds **1–9**) depicted in Figure 1. In these conjugates, the PA chain is either directly introduced into the 3-position of the propane-1,3-diol backbone of CAM or via a dicarboxylic acid linker replacing the dichloroacetyl tail of CAM. With these particular conjugates we wanted to examine how the size of the PA chain and the number of its free amino functions (e.g. compounds **1–3**), the lipophilicity of the PA chain (e.g. compounds **3** and **4**), the nature and flexibility of the linker (e.g. compounds **1** and **6**), the site of the PA chain attachment on CAM (e.g. compounds **2** and **7**), and inversely the site of the CAM attachment on the PA chain (e.g. compounds **4** and **5**), can influence the antibacterial and anticancer properties of the constructs. Finally, we included in this study two derivatives of CAM in which the dichloroacetyl part of the molecule was replaced by the 1,2,4-triazole-3-carboxylate unit, which was either directly connected to the 2-amino group (amide **8**) or through a β-alanine spacer (bisamide **9**). Through these compounds we investigated the effect caused by replacing the two chlorine atoms of CAM by N atoms and evaluated whether removing this replacement away from the 2-aminopropane-1,3-diol main chain would have any effect on the activity of the constructs. The mechanism of binding of the synthesized PA–CAM conjugates to *E. coli* ribosomes was investigated by kinetic and time-resolved footprinting analysis, while their antibacterial activities were tested against wild-type strains of *E. coli* and *Staphylococcus aureus*, and against CAM-resistant mutants of *E. coli.* Finally, we studied the effect of PA–CAM conjugates on the viability of human peripheral blood cells, human leukemic cells and other cancer cell lines. Our results show that some of the PA–CAM conjugates can be used as lead compounds for designing new drugs with improved antibacterial and anticancer properties.

## MATERIALS AND METHODS

### Materials, bacterial strains and cell lines

CAM free base [d-(-)*threo*-1-(*p*-nitrophenyl)-2-amino-1,3-propanediol], puromycin dihydrochloride, tRNA^Phe^ from *E. coli*, dimethyl sulfate (DMS), DMS stop solution and tRNA^Phe^ from *E. coli* were purchased from Sigma-Aldrich. Kethoxal and 1-cyclohexyl-3-(2-morpholinoethyl)-carbodiimide metho-p-toluene sulfate (CMCT) were from MP Biomedicals and Fluka Biochemicals, respectively. AMV reverse transcriptase was supplied by Roche, dNTPs by HT Biotechnology, and ddNTPs by Jena Bioscience. l-[2,3,4,5,6 -^3^H]Phenylalanine was from Amersham Biosciences and [α-^32^P]ATP from Izotop. Cellulose nitrate filters (type HA; 0.45 μm pore size) were from Millipore Corp. Details in experimental procedures of synthesis and physical and spectra data for the synthesized compounds will be published elsewhere. *E. coli* TA531 cells lacking chromosomal *rrn* alleles, but containing pKK35 plasmids possessing wild-type or mutated 23S rRNA (A2058G or A2503C), were kindly offered by Dr A.S. Mankin (University of Illinois). The mesothelioma cell line ZL34 and its immortalized counterpart cell line Met5A, were kindly provided by Prof. G. Stathopoulos (University of Patras).

### Biochemical preparations

Isolation of 70S ribosomes from *E. coli* K12 cells and preparation of Ac[^3^H]Phe-tRNA^Phe^ charged to 80% were performed, as described previously (23). The post-translocation complex of poly(U)-programmed ribosomes (complex C), bearing tRNA^Phe^ at the E-site and Ac[^3^H]Phe-tRNA at the P-site was prepared in buffer A (100 mM Tris-HCl pH 7.2, 6 mM (CH_3_COO)_2_Mg, 100 mM NH_4_Cl and 6 mM 2-mercaptoethanol). The percentage of active ribosomes in AcPhe-tRNA binding was 72%. This ribosomal population was more than 90% reactive toward puromycin.

### Sensitivity to CAM and PA–CAM conjugates of *S. aureus* and *E. coli* cells containing wild-type or mutant ribosomes

*S. aureus* or *E. coli c*ells (400 μl of a 0.700 OD_560_ preculture) containing wild-type or mutant ribosomes were added in 3.6 ml of LB (Luria Broth) medium and grown at 37°C in the presence or absence of CAM or PA–CAM conjugates until the optical density of the control culture (grown in the absence of drug) reached the value 0.700 at 560 nm. From the growth curves, the IC_50_ value for each strain was estimated as the concentration of the drug that is required to inhibit the growth by half.

### Toxicity assays in human peripheral blood cells and leukemic cell lines

Peripheral blood was collected in EDTA-coated tubes from five healthy volunteers (age range: 25–30 years). Cell concentration was adjusted to 1.8 × 10^9^ cells/l using RPMI-1640 medium (GIBCO BRL) containing 1% Penicillin/Streptomycin. CAM or PA–CAM conjugates were added at various concentrations and cells were cultured in triplicate under a humidified 5% CO_2_ atmosphere for 5 days, at 37°C. In parallel, cells were cultured in the absence of CAM or PA–CAM conjugates (control cultures). Counting of cells was performed daily in a CELL-DYN 3700 Hematology Analyzer (Abbott, USA) and values were expressed as a percentage of cells measured in control cultures.

Human leukemic cell lines, HS-Sultan (Burkitt's lymphoma) (European Collection of Cell Cultures, Salisbury, UK), Jurkat (T-cell acute lymphoblastic leukemia) and U937 (histiocytic lymphoma) (American Type Culture Collection, Manassas, USA), were adjusted to 1 × 10^9^ cells/l in RPMI-1640 medium containing 1% Penicillin/Streptomycin and 10% fetal bovine serum and grown in triplicate in the presence or absence of CAM or PA–CAM conjugates for 4 days at 37°C, under a humidified 5% CO_2_ atmosphere. The medium was changed after 2 days of exposure; CAM or PA-CAM addition was repeated after medium change to keep the appropriate drug concentration. Aliquots were collected daily and counted in a CELL-DYN 3700 Hematology Analyzer. For cell necrosis and apoptosis assays, samples (10^6^ cells) were collected daily and determined using the Annexin V-PE Apoptosis Detection Kit I (BD Pharmingen) for flow cytometry (29), as described by the manufacturer. Flow cytometry data were analyzed using the FlowJo flow cytometry analysis software. Necrotic and apoptotic cells were expressed as a percentage of total cells.

### Treatment of ZL34 and Met5A cell lines with CAM or PA–CAM conjugates

ZL34 and Met5A cells were plated in sterile 96-well microtiter plates at 5 × 10^4^cells/ml and grown in Dulbecco's modified Eagle's medium (DMEM), provided by Sigma-Aldrich and supplemented with 5% fetal bovine serum. Cultures were maintained in a humidified atmosphere with 5% CO_2_, at 37°C. Solutions at the appropriate concentration of each compound were added, and then cells were grown for 24, 48, 72 and 96 h. After treatment, the drug was removed by washing the cells twice with phosphate buffered saline. The cells were then trypsinized (100 μl Trypsin-EDTA × 1 (Biosera) solution/well, 10 min at 37°C), mixed with 1 ml DMEM and collected by centrifugation at 3000 × *g* for 5 min. Cell viabilities were determined by the trypan blue exclusion assay, using a TC10 automated cell counter (BIO-RAD). Viable cells were expressed as a percentage of total cells.

### Inhibition of peptide bond formation by CAM or PA–CAM conjugates

The reaction between complex C and excess puromycin (S), a pseudo-substrate which binds to the ribosomal A-site, was performed at 25°C in buffer A and analyzed as described in detail elsewhere (11). Briefly, since the reaction followed first-order kinetics, the first-order rate constant *k*_obs_ at each concentration of puromycin was determined by fitting the corrected *x*-values into Equation (1),

(1) \begin{equation*} \ln \frac{{100}}{{100 - {x}}} = k_{{\rm obs}} t \end{equation*}

where *x* is the product Ac[^3^H]Phe-puromycin expressed as the percentage of complex C radioactivity added in the reaction mixture and *t* the time of the reaction. *k*_obs_ is related to the puromycin concentration, [S], by the relationship,

(2) \begin{equation*} k_{{\rm obs}} = \frac{{k_{{\rm cat}} [{\rm S}]}}{{K_{\rm S} + [{\rm S}]}} \end{equation*}

where *k*_cat_ is the catalytic rate constant of PTase and *K*_S_ the affinity constant of puromycin for complex C.

In the presence of CAM–polyamine conjugates, biphasic logarithmic time plots were obtained. The slope of the straight line through the origin was taken as the value of the apparent rate constant, *k*_ob*s*(early)_, at the early phase of the reaction, while the slope of the second straight line was taken as the value of the rate constant, *k*_obs(late)_, at the late phase of the reaction.

### Time-resolved binding of PA–CAM conjugates to *E. coli* ribosomes

Ribosomes (R) from *E. coli* (100 nM) were incubated either alone or with a PA–CAM conjugate at concentration equal to 50 × *K*_i_ in 100 μl of buffer B [HEPES/KOH, pH 7.2, 10 mM Mg(CH_3_COO)_2_, 100 mM NH_4_Cl, and 5 mM dithiothreitol] at 25°C, either for 2 s (RI probing) or for longer than 10 × *t*_1/2_ min (R*I probing). The term *t*_1/2_, which represents the half-life for the attainment of equilibrium between ribosomes and each conjugate, was estimated through the relationship,

(3) \begin{equation*} t_{1/2} = \frac{{0.693}}{{k_{{\rm eq}} }} \end{equation*}

where *k*_eq_ is the apparent equilibration rate constant, given by Equation (4).

(4) \begin{equation*} k_{{\rm eq}} = k_{{\rm off}} + k_{{\rm on}} \frac{{[{\rm I}]}}{{K_{\rm i} + [{\rm I}]}} \end{equation*}

Complexes RI and R*I were then probed at 37°C for 10 min with DMS, kethoxal or CMCT, as described previously (30). The modified sites in 23S rRNA were then analyzed by primer extension with reverse transcriptase, according to Moazed et al. (30). Since previous studies have localized the footprints of CAM within the PTase center and the entrance to the exit tunnel, the primers were complementary to the sequences 2102–2119, 2561–2578 and 2680–2697 of 23S rRNA, provided that the size of PA–CAM conjugates does not exceed 30 Å. Extension products were run on 6% polyacrylamide/7M urea gels. Identification of the modified nucleotides, quantitative scanning of the gels and normalization of the band intensities were made as previously described (4). The values indicated in Table 2 denote the ratio between the normalized intensity of a band of interest and the normalized intensity of the corresponding band in the control lane (ribosomes non-treated with drugs).

**Table 1.** Equilibrium and kinetic constants involved in the inhibition of AcPhe-puromycin synthesis by CAM and PA–CAM conjugates^a^

PA–CAM conjugates	Constant	*K*_i_ (μM)	*K*_ι_* (μM)	*k*_on_/*k*_off_	*k*_on_ (min^−1^)	*k*_off_ (min^−1^)
**CAM**^b^		3.10 ± 0.30	0.88 ± 0.07	2.52 ± 0.44	2.29 ± 0.13	0.99 ± 0.04
**1**		3.37 ± 0.30	0.90 ± 0.06	2.74 ± 0.42	2.88 ± 0.10	1.05 ± 0.10
**2**		2.20 ± 0.20	0.75 ± 0.06	1.93 ± 0.36	2.30 ± 0.10	1.19 ± 0.16
**3**		3.60 ± 0.30	1.20 ± 0.10	2.00 ± 0.35	2.49 ± 0.12	1.26 ± 0.13
**4**		0.98 ± 0.08	0.28 ± 0.03	2.48 ± 0.42	2.10 ± 0.12	0.66 ± 0.09
**5**		2.10 ± 0.17	0.60 ± 0.05	2.50 ± 0.40	2.22 ± 0.16	0.89 ± 0.08
**6**		214.00 ± 17.12	8.45 ± 0.67	24.32 ± 2.85	18.24 ± 1.21	0.75 ± 0.06
**7**		16.25 ± 1.46	5.00 ± 0.45	2.25 ± 0.41	2.89 ± 0.21	1.28 ± 0.15
**8**		3.00 ± 0.30	1.00 ± 0.10	2.0 ± 0.35	2.19 ± 0.17	1.10 ± 0.12
**9**		12.0 ± 1.08	4.00 ± 0.44	2.0 ± 0.42	1.67 ± 0.18	0.84 ± 0.08

^a^Data denote the mean ±S.E. values obtained from three independently performed experiments, with two replicates per experiment.

^b^Data taken from (11).

**Table 2.** Relative reactivity of nucleosides in the central loop of Domain V of 23S rRNA, when a PA–CAM conjugate (I) binds *E. coli* ribosomes (R) at the initial (RI) and the final (R*I) binding site^a^

23S rRNA residue		Compound **4**			Compound **5**		
	Binding state	R	RI	R*I	R	RI	R*I
A2058		1	1.00 ± 0.05	1.25 ± 0.09^b,c^	1	1.10 ± 0.09	0.95 ± 0.08
A2059		1	1.00 ± 0.06	0.60 ± 0.03^b,c^	1	0.90 ± 0.08	1.00 ± 0.08
A2062		1	1.00 ± 0.05	0.52 ± 0.02^b,c^	1	1.00 ± 0.06	1.00 ± 0.09
A2451		1	0.48 ± 0.02^b^	0.53 ± 0.05^b^	1	0.65 ± 0.04^b^	0.70 ± 0.05^b^
G2505		1	0.65 ± 0.05^b^	0.35 ± 0.02^b,c^	1	0.74 ± 0.06^b^	0.50 ± 0.15^b,c^
U2506		1	0.70 ± 0.05^b^	0.65 ± 0.04^b^	1	0.75 ± 0.05^b^	0.70 ± 0.06^b^
U2585		1	0.80 ± 0.07^b^	0.45 ± 0.02^b,c^	1	0.92 ± 0.10	0.80 ± 0.10^b^
A2602		1	1.00 ± 0.10	0.88 ± 0.08	1	1.00 ± 0.06	0.89 ± 0.07
U2609		1	1.00 ± 0.09	1.00 ± 0.07	1	1.00 ± 0.10	0.90 ± 0.08

^a^Relative reactivity of nucleosides denotes the ratio between the normalized intensity of a band of interest and the normalized intensity of the homologous band in the control lane (R). Only nucleosides that are accessible to DMS, CMCT and kethoxal are indicated, while their accessibility changes upon exposure to a PA–CAM conjugate.

^b^Significantly different in relation to R (*P* < 0.05).

^c^Significantly different in relation to RI (*P* < 0.05).

### System modeling and molecular dynamics simulations

Three dimensional (3D) models of compounds **4** and **5** were achieved using Arguslab 4.0.1 provided by Planaria Software LLC, Seattle, WA (http://www.arguslab.com), starting with the 3D structure of CAM derived from the 50S ribosomal subunit structure of *E. coli* in complex with CAM (31; PDB:3OFC). CHARMM Force field parameters and topology files were generated by the SwissParam Tool (32). The PA-CAM molecules were docked into the 50S ribosomal subunit structure, by positioning their CAM moiety within the drug pocket as indicated in (31 ). All groups of 50S subunit in a distance of 10 Å around compounds **4** and **5**, except for water, were selected, solvated with TIP3 water molecules, and then neutralized with sodium ions using the VMD program (33). The systems produced in this way are referred hereafter as rib-4 and rib-5, respectively. For comparative purposes, a similar system was prepared for CAM itself. This system is referred as rib-CAM.

Rib-4, rib-5 and rib-CAM were energy minimized and subjected to canonical enseble Molecular Dynamics (MD) simulations for 10 ns at 300K, with Particle Mesh Ewald (PME) algorithm and rigid bonds assigned using the NAMD software (34). During MD simulations, all nucleic acid backbone atoms were immobilized. Finally, the last frame of each of the three MD trajectories was energy minimized. All molecular visualizations were produced with the PyMOL Molecular Graphics System, Version 1.5.0.4 Schrödinger, LLC.

### Statistics

All data presented in the Figures and Tables denote the mean values obtained from three independently performed experiments, with two replicates per experiment, estimated by one-way ANOVA. Statistical tests (data variability, *F*-Scheffé test) were performed using the program IBM Statistics 19.

## RESULTS

### PA–CAM conjugates act on the ribosome as slow-binding, competitive inhibitors of peptide-bond formation

**Figure 2.**
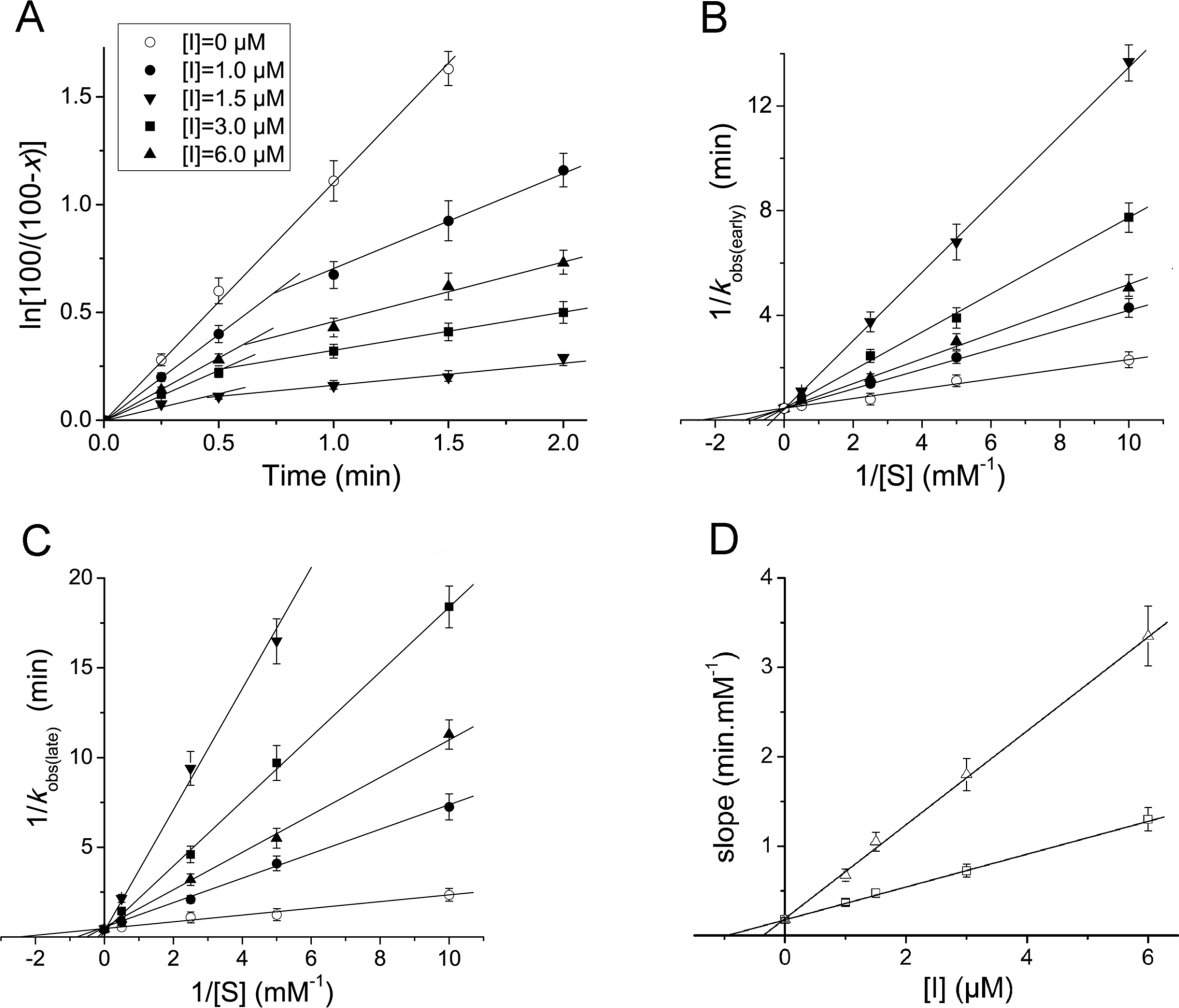
Kinetic plots for the AcPhe-puromycin synthesis in the presence or absence of PA–CAM conjugate **4**. (**A**) First-order time plots; complex C reacted at 25°C in buffer A, with (open circles) 400 μM puromycin or with a mixture containing (total concentration) 400 μM puromycin and compound **4** at (filled circles) 1 μM, (up-standing, filled triangles) 1.5 μM, (filled squares) 3 μM and (down-standing, filled triangles) 6 μM. The deviation from linearity observed in the presence of compound **4** reveals a time-dependent inhibition effect. (**B** and **C**) Double-reciprocal plots; the data shown were collected from the early and the late phases of semi-logarithmic plots, respectively, such as those presented in panel A. Drug concentrations are denoted by the same symbols, as those used in panel A. (**D**) Slope replots (slopes of the double-reciprocal plots versus compound **4** concentration). The slope values were estimated from the plots shown in (open squares) panel B or (open triangles) panel (C). The plots presented in panels B, C and D indicate that the inhibition at both phases is of competitive type and that only one ribosomal binding site is implicated at each phase of the inhibition process.

**Scheme 1.**
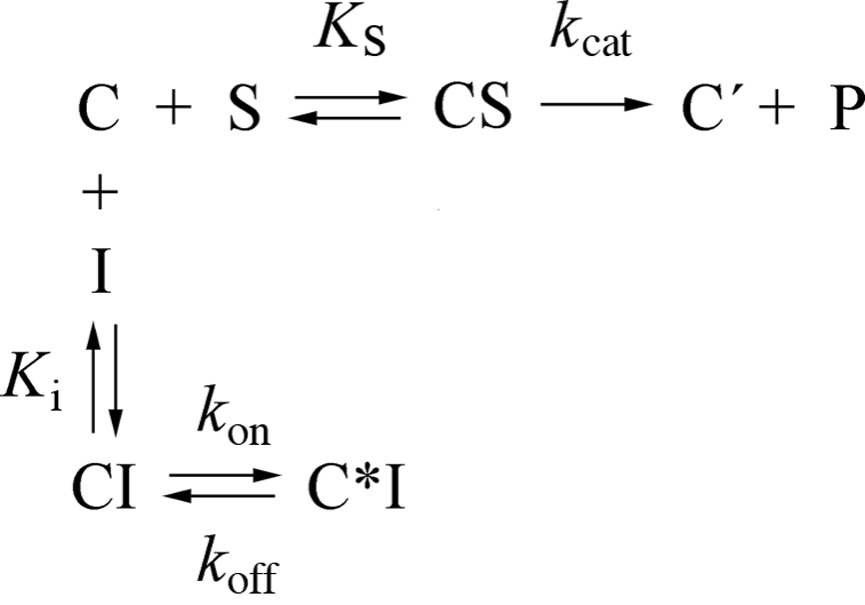
Kinetic model for the inhibition of the puromycin reaction by compound **4**. Symbols: C, poly(U)-programmed ribosomes from *E. coli* bearing Ac[^3^H]Phe-tRNA^Phe^ at the P-site, and tRNA^Phe^ at the E-site; S, puromycin; P, Ac[^3^H]Phe-puromycin; C′, ribosomal complex not recycling; I, compound **4**.

The reaction between complex C, a model post-translocation complex of poly(U)-programmed ribosomes bearing tRNA^Phe^ at the E-site and Ac[^3^H]Phe-tRNA at the P-site, and puromycin in excess proceeds under single turnover conditions. Therefore, it displays pseudo-first-order kinetics. Consistently, the semi-logarithmic time plot, *ln*[100/(100-*x*)] versus *t*, is represented by a straight line. A representative plot obtained at 400 μM puromycin is shown in Figure 2A (upper line). However, when the puromycin reaction is carried out in the presence of compound **4**, two phases can be clearly seen, the first one proceeding much faster than the second one (Figure 2A, four lower lines). Moreover, both phases exhibit characteristics of competitive inhibition, while the initial slope of progress curves varies as a function of the compound **4** concentration (Figure 2A,B,D). This inhibition pattern is reminiscent of those previously observed for CAM (11). Therefore, we assumed that the kinetic scheme adopted for the CAM mechanism of action (Scheme 1) could adequately explain the mode of action of compound **4**.

**Figure 3.**
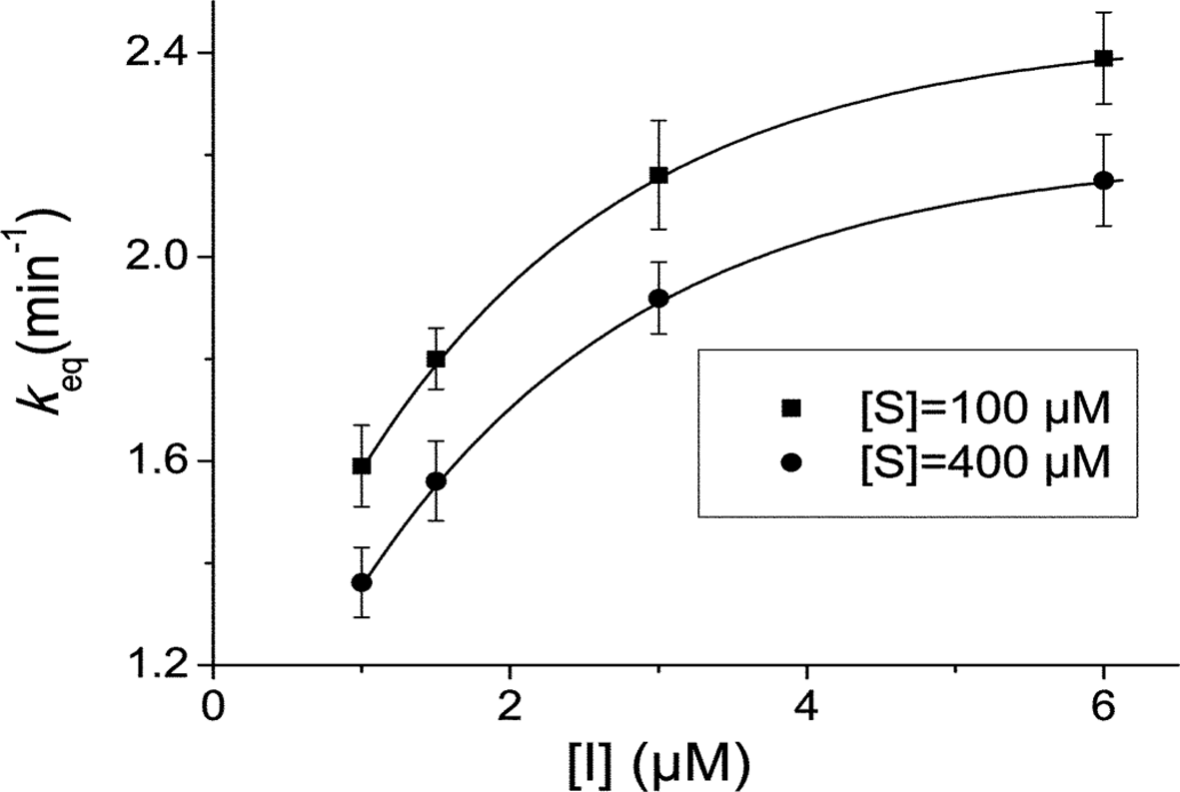
Variation of the apparent equilibration rate constant, *k*_eq_, as a function of compound **4** concentration (I). The reaction was carried out in buffer A, in the presence of puromycin at (filled squares) 100 μM or (filled circles) 400 μM. The *k*_eq_ values were determined by non linear regression fitting of the kinetic data to Equation (5). The hyperbolic character of plots denotes that compound **4** inhibits the puromycin reaction by a two-step mechanism.

**Figure 4.**
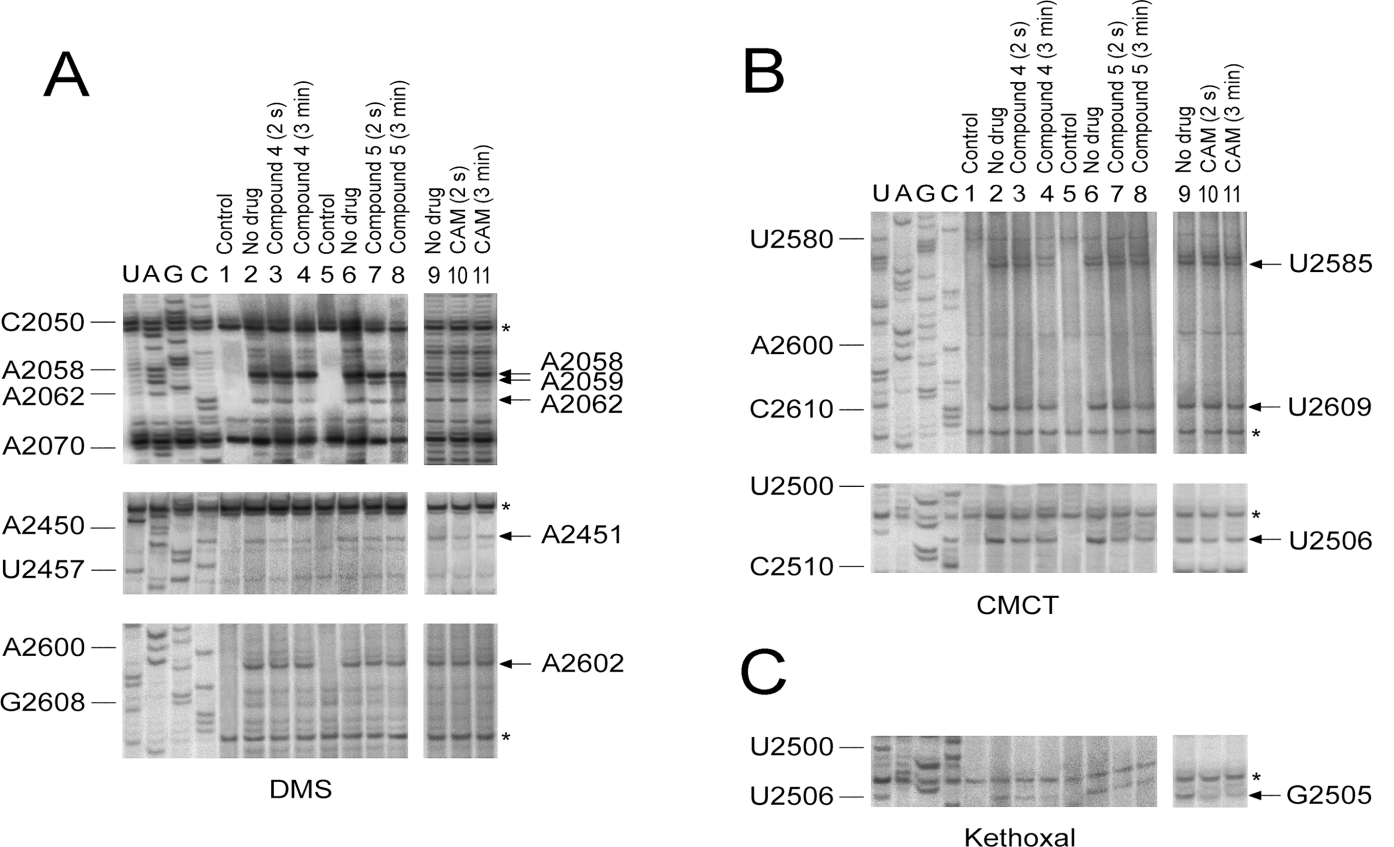
Protections against chemical probes in nucleotides of the central loop of domain V of 23S rRNA, caused by binding of PA–CAM conjugates (compounds **4** and **5**) to *Escherichia coli* ribosomes. Ribosomes were incubated in the presence or absence of each PA–CAM conjugate at 25°C for 2 s or 3 min. The resulting complexes were then probed with DMS (**A**), CMCT (**B**) or Kethoxal (**C**). U, A, G and C, dideoxy sequencing lanes; lanes 1 and 5, unmodified ribosomes; lanes 2 and 6, ribosomes probed in the absence of PA–CAM conjugates; lanes 3 and 7, ribosomes pre-incubated with each PA–CAM conjugate for 2 s and then probed; lanes 4 and 8, ribosomes pre-incubated with each PA–CAM conjugate for 3 min and then probed. Results obtained with CAM, although published previously (4), were reproduced, and are presented in lanes 9–11 for the sake of comparison. Numbering of nucleosides for the sequencing lanes is indicated at the left. Nucleosides with accessibility affected by bound PA–CAM conjugate are shown by arrows at the right, while reference bands whose intensity is not affected by PA–CAM conjugates or CAM binding are indicated by an asterisk. The relative intensity of each reference band between lanes was used to correct the variability between lanes (horizontal normalization).

**Figure 5.**
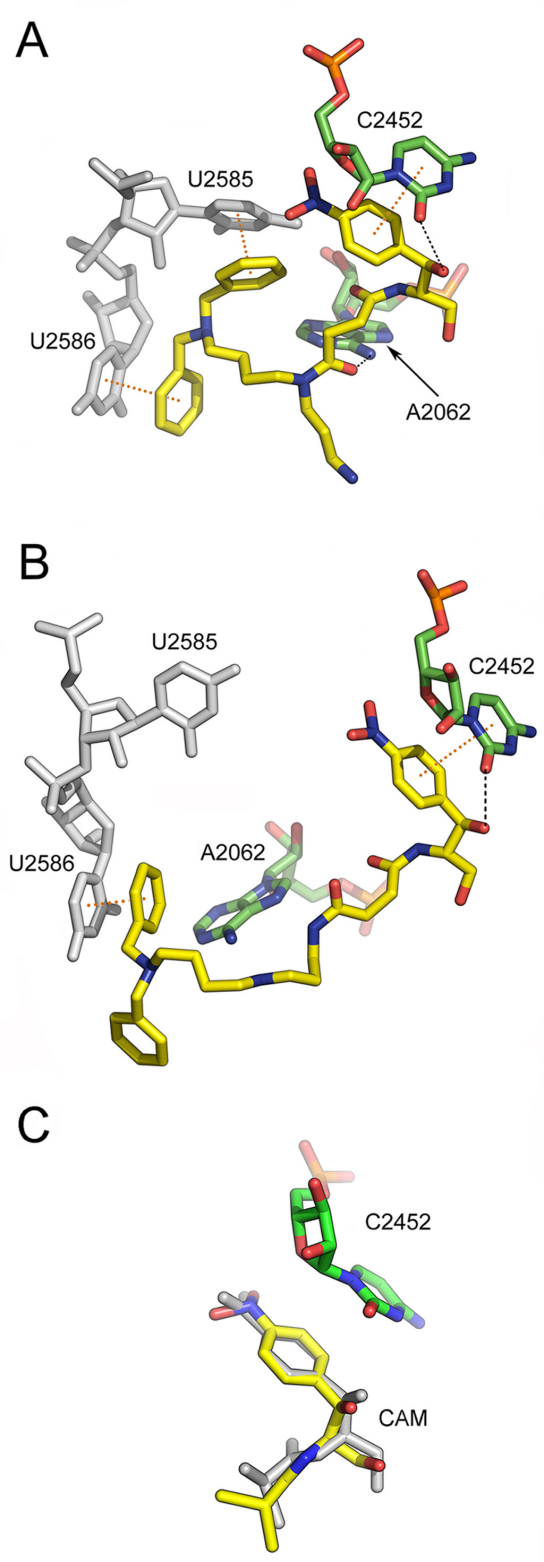
Binding position of two PA–CAM conjugates on the *Escherichia coli* ribosome, as detected by Molecular Dynamics Simulations. The ligand models have been docked into the 50S ribosomal subunit, by positioning their CAM moiety within the CAM crystallographic pocket (31). (**A**) Binding position of compound **4** (yellow); hydrogen bonding with A2062 and C2452 is shown by black dashes, while *π*-stacking arrangements with U2585 and U2586 (gray) are indicated by yellow dots connecting the centers of the interacting aromatic rings. Other residues of 23S rRNA placed adjacently to the binding pocket of **4** are ignored for clarity. (**B**) Binding position of compound **5** (yellow); a *π*-stacking interaction of **5** with U2586 (gray) is shown at the left. (**C**) Overlay of CAM structures from MD simulation (yellow) and crystallographic analysis (gray; PDB:3OFC). Other residues of 23S rRNA placed adjacently to the binding pocket of CAM, except for C2452, are ignored for clarity.

According to Scheme 1, compound **4** (I) binds to complex C (C) to form instantaneously the encounter complex CI, which is then isomerized slowly to form a tighter complex, C*I. Supporting evidence for the consistency of this model is provided by the plots of *k*_eq_ versus [I] that are hyperbolic (Figure 3). If one-step mechanism was applicable, the equilibration plots should be linear (4,35). It should be mentioned that the apparent equilibration rate constant *k*_eq_ can be estimated from the intersection point of the two linear parts of progress curves, like those illustrated in Figure 2A; at this point, *k*_eq_ = 1/*t*. However, the intersection points cannot be precisely localized. Therefore, the *k*_eq_ values were determined by non linear regression fitting of the kinetic data to Equation (5),

(5) \begin{eqnarray*} &&\ln \frac{{100}}{{100 - x}} = \nonumber \\ &&k_{{\rm obs}({\rm late})} t + \frac{{\left[ {k_{{\rm obs}({\rm early})} - k_{{\rm obs}({\rm late})} } \right]}}{{k_{{\rm eq}} }}(1 - e^{k_{{\rm eq}} t} ) \end{eqnarray*}

which holds for slow-binding inhibitors (11).

The straight lines shown in Figure 2D, when extrapolated, meet the horizontal axis of the plot at a point pertaining to the inhibition constant, which is for the early phase (*K*_i_) and the late phase of the reaction (*K*_i_*) equal to 0.975 ± 0.080 μM and 0.280 ± 0.030 μM, respectively. As previously indicated (11), *K*_i_* is related to *K*_i_ by the relationship (6).

(6) \begin{equation*} K_{\rm i}^* = k_{\rm i} \frac{{k_{{\rm off}} }}{{k_{{\rm on}} + k_{{\rm off}} }} \end{equation*}

Therefore, the isomerization constant *k*_on_/*k*_off_ can be calculated through this relationship. We found that *k*_on_/*k*_off_ for compound **4** equals 2.48 ± 0.30. The individual values of *k*_on_ and *k*_off_ were calculated by non linear regression fitting of the kinetic data to Equation (7),

(7) \begin{equation*} k_{{\rm eq}} = k_{{\rm off}} + k_{{\rm on}} \frac{{\frac{{[{\rm I}]}}{{K_{\rm i} }}}}{{1 + \frac{{[{\rm S}]}}{{K_{\rm S} }} + \frac{{[I]}}{{K_{\rm i} }}}} \end{equation*}

which holds when the interaction between complex C and the inhibitor is carried out in the presence of puromycin (11). These values are presented in Table 1.

Interestingly, all the other compounds shown in Figure 1 exhibited the same kinetic pattern adopted by compound **4**. The values of the kinetic parameters concerning these compounds are depicted in Table 1. Evidently, compounds **4** and **5** are ranking among the most potent members in the group of PA–CAM conjugates in inhibiting the puromycin reaction, even exceeding the potency of CAM.

### Time-resolved footprinting analysis confirms the stepwise binding of PA–CAM conjugates to *E. coli* ribosomes and allows the characterization of the ribosomal binding sites implicated in each step

Adopting a time-resolved footprinting approach, recently applied in dissecting the interactions of various slow-binding inhibitors of PTase with ribosomes derived from *E. coli* cells (4,36–38), footprinting of complex RI was achieved by incubating *E. coli* ribosomes (R) with compounds **4** or **5** in buffer B, at 25°C for 2 s. Each compound was added to the incubation mixture at concentration equal to 50 × *K*_i_, thus allowing most of ribosomes to exist in complex with the compound. Substrate-free ribosomes were used instead of complex C, to ensure that the ribosomal population is homogeneous and avoid protection effects caused by tRNAs bound to the ribosome. Because the first step of binding, R + I ⇄ RI, attains to equilibrium instantaneously while the second step, RI ⇄ R*I, proceeds slowly, the main ribosomal species existing at the end of this time interval is complex RI (>88%). Taking into account that the chemical probes subsequently used react with accessible nucleosides within a few milliseconds (5), the footprinting pattern that was achieved concerns complex RI. However, when ribosomes and the conjugate were incubated for 3 min corresponding to more than 10 half lives (10 × *t*_1/2_), most of the ribosomes (>70%) at the end of this time interval were in the R*I binding state, provided that the value of the isomerization constant, *k*_on_/*k*_off_, equals 2.5 (Table 1). Therefore, the footprinting pattern observed after incubation of each compound with ribosomes for 3 min corresponds to complex R*I.

Representative autoradiograms obtained by primer extension analysis of the probed complexes are shown in Figure 4 and the relative intensities of the bands of interest are summarized in Table 2. As indicated in Table 2, compound **4** at the RI binding state strongly protects nucleosides A2451, G2505 and U2506, and faintly U2585. After longer exposure of ribosomes to compound **4**, the previously observed protective effects on G2505 and U2585 were potentiated, new protections appeared on nucleosides A2062 and A2059, whereas the accessibility of A2058 to DMS was slightly enhanced (see also Figure 4). The footprinting pattern of compound **5** at the RI binding state resembled that obtained with compound **4**, except that the protections seen in nucleosides A2451, G2505 and U2506 slightly softened. Larger differences, however, were observed when compound **5** was at the R*I binding state; effects on A2058, A2059 and A2062 to DMS were abolished, while the protection effect on U2585 was strongly reduced.

### PA–CAM conjugates 2–5 and 9 are effective in inhibiting the growth of bacterial cell cultures

Wild-type *S. aureus* and *E. coli* cells were used as model strains of Gram-positive and Gram-negative bacteria, respectively. Two mutants of *E. coli* lacking chromosomal *rrn* alleles, but containing pKK35 plasmids possessing A2058G or A2503C mutations in 23S rRNA, known to be related with decreased sensitivity against CAM (15,39), were used to reveal effects of PA–CAM conjugates on a CAM-resistance phenotype. As shown in Table 3, none of PA–CAM conjugates was better than CAM in inhibiting the growth of wild-type *S. aureus* or *E. coli* cells. Nevertheless, compounds **2–5** and **9** had antibacterial activity with IC_50_ values at micromolar range. More importantly, mutations A2058G and A2503C failed to provide any growth advantage to *E. coli* cells against compounds **4** and **5**, similar to that conferred against CAM. Especially, compound **4** maintained ∼2-fold IC_50_ superiority over CAM, in inhibiting these mutants. By analyzing the relationship between antimicrobial activities of PA–CAM conjugates and their inhibitory properties in peptide-bond formation, it is evident that the ratio IC_50_/IC_50(puro)_ for wild-type bacteria, in which IC_50(puro)_ is defined as the conjugate concentration causing 50% inhibition in peptide-bond formation at 2 mM puromycin, is much lower for CAM than for any PA–CAM compound (Supplementary Table S1), a fact revealing that penetration of the cellular envelope may be a significant obstacle to the effectiveness of PA–CAM conjugates acting as antibiotics.

**Table 3.** Determination of the half maximal concentration, IC_50_, for CAM and PA–CAM conjugates, indicating how much of each compound is needed to inhibit the growth of wild-type *S. aureus* and *E. coli* cells or *E. coli* mutants by half^a,b^

Compound	IC_50_ (μM)			
	WT-*S. aureus*	WT-*E. coli*	*E. coli* (A2058G)	*E. coli* (A2503C)
**CAM**	3.1 ± 0.3	6.2 ± 0.5	15.5 ± 1.3	24.7 ± 2.3
**1**	>200	>200	>200	>200
**2**	45.3 ± 5.5	>100	>100	>100
**3**	12.7 ± 1.0	>150	>150	>150
**4**	4.7 ± 0.5	9.4 ± 0.8	9.4 ± 1.0	9.4 ± 0.9
**5**	13.7 ± 1.2	35.5 ± 3.6	32.3 ± 3.0	37.1 ± 3.1
**6**	>200	>200	>200	>200
**7**	>200	>200	>200	>200
**8**	>100	>300	>300	>300
**9**	66.0 ± 4.6	>200	>200	>200

^a^Data represent the mean ± SE values obtained from three independently performed experiments, with two replicates per experiment

^b^*E. coli* TA531 cells lacking chromosomal *rrn* alleles, but containing pKK35 plasmids possessing wild-type 23S rRNA displayed a similar IC_50_ value for each compound, to those of wild-type (WT) *E. coli* K12 cells.

### Cytotoxicity of CAM and compound 4 against human peripheral blood cells and leukemic cell lines

Taking into account the adverse affects of CAM on the bone marrow cells (20), the efficacy of compound **4** as a safe antibacterial agent was tested against human peripheral blood cells and leukemic cell lines. CAM was used as a reference compound. CAM, and to a lesser degree compound **4**, caused a 20% reduction in the viability of neutrophils, when added to the culture medium at a concentration of 30 μM for 24 or 48 h; effects on other types of leukocytes were negligible (Supplementary Figure S1).

To determine the effect of compound **4** on leukemic cell lines, HS-Sultan, Jurkat and U937 cells were treated with increasing concentrations of this compound. CAM was used as a reference compound. Preliminary experiments, carried out by counting the cells daily in a CELL-DYN 3700 Hematology Analyzer, showed that HS-Sultan and Jurkat cells were sensitive to both agents, while U937 cells were insensitive to compound **4** (Supplementary Figure S2). For this reason, the effect of compound **4** on HS-Sultan and Jurkat cells was further investigated by flow cytometry. The results showed that compound **4** at 60 μM induced necrosis to both HS-Sultan and Jurkat cells, and apoptosis to HS-Sultan cells. In comparison, CAM at 60 μM induced necrosis to HS-Sultan cells, but failed to induce apoptosis to both cell lines (Supplementary Figures S3 and S4).

### Cytotoxicity of CAM and compound 4 against human cancer cells

Compound **4** exhibited good *in vitro* selectivity in targeting human mesothelioma cells ZL34 (IC_50_ = 25 ± 3 μM) and immortalized human mesothelial Met5A cells (IC_50_ = 60 ± 5 μM) (Supplementary Figure S5). CAM showed no selectivity and low toxicity for both cell lines with an IC_50_ higher than 300 μM. Other PA-CAM compounds displayed lower or no toxicity against these cells. Remarkably, exogenously added spermidine at 10 × IC_50_ was able to significantly rescue (back to ∼95% viability) ZL34 cells exposed to an IC_50_ dose of compound **4**, whereas Met5A cells recover little of the lost viability. When 3 mM spermidine was added to ZL34 cells treated with 300 μM CAM, no rescue was observed. We rationalized these findings as being due to the relative propensity of compound **4** to use the polyamine transport system for ZL34 cell entry.

## DISCUSSION

In the present study, we examined the inhibition of peptide bond formation by a series of PA–CAM conjugates. The rationale for the synthesis of these compounds was to explore the properties of the positively charged ammonium cations and/or benzyl groups synthetically incorporated into the polyamine backbone, which would provide additional interactions with ribosomal regions surrounding the CAM binding site in the PTase center. In an alternative approach, we envisioned that conjugation of CAM with PAs might be an effective way of selectively delivering CAM into human cancer cells, taking advantage of the upregulated polyamine transport system of these cells (27,40), and then selectively targeting mitochondria (41,42).

PA–CAM conjugates interacted with complex C with an apparent association rate constant, (*k*_on_ + *k*_off_)/*K*_i_, that was lower than 10^6^ M^−1^ s^−1^, which has been set as the upper limit for the characterization of a ligand as slow-binding inhibitor (35). The slow binding of PA–CAM conjugates into the PTase center and the slow dissociation from it are of high biological significance, because they provide time for conformational ribosome-inhibitor adjustments, such as induced fit (43). On the other hand, the slow *k*_off_ rate provides long residence time for each PA–CAM conjugate at its target, which has been widely accepted as a good predictor of the *in vivo* drug efficacy (44).

In a closed *in vitro* protein-synthesizing system, like the one we use in our study, ribosome and an inhibitor are at equilibrium, and thus equilibrium constants, such as *K*_i_, could be appropriate metrics for differentiating inhibitor potency (44). However, the potency of each PA–CAM conjugate cannot be assigned solely on the basis of *K*_i_ alone, given that the equilibrium between complex C and the conjugate is attained via a two-step mechanism. Therefore, we propose the use of *K*_i_*k*_7_/(*k*_6_ + *k*_7_) formula, because it represents the overall tendency of each conjugate for engagement in both sequential reactions of the kinetic model. Accordingly, we estimated that compounds **4** and **5** are 3-fold and 1.5-fold, respectively, more potent than CAM. The other PA–CAM conjugates exhibit equal (compounds: **1**, **2** and **8**) or lower potency (compounds: **3**, **6**, **7** and **9**) than that calculated for CAM. Noteworthy, benzyl substitution of the *N*8-amino group of spermidine moiety in compound **4** has a significant impact on both the recognition of compound **4** by complex C (low *K*_i_) and the stability of the final complex C*I (low *k*_off_) (compare compounds **3** and **4**). Moreover, the spatial placement of the benzylated amino group of PA relatively to the CAM scaffold is critical for the functional accommodation of the conjugate within the catalytic center of the ribosome (compare compounds **4** and **5**). The position of the CAM scaffold, through which the PA is linked, is of paramount importance; connection of PA to the 3-position of the 2-aminopropane-1,3-diol moiety of CAM, instead of the dichloroacetyl tail, leads to a strong reduction of potency as well as to an enhancement of the *k*_off_ value (compare compounds **2** and **7**). This is in agreement with previous studies indicating that the conformation and integrity of the 2-aminopropane-1,3-diol portion of CAM are both essential for the activity of the drug (22,45). The design of compound **7** was based on X-ray crystallographic observations in *Deinococcus radiodurans* 50S ribosomal subunit complexed with CAM (46), according to which the primary hydroxyl group of CAM interacts with the *O*4 of U2506 (*E. coli* numbering is used throughout the text), through a Mg^2+^ ion that coordinates both groups. Nevertheless, the activity of compound **7** in our study was found much lower than expected (Table 1). In fact, this crystallographic model (46) got the orientation of CAM completely wrong, as mentioned in recent models of CAM complexed with *E. coli* or *Thermus thermophilus* ribosomes (31, 53), and the polyamine group added in compound **7** would therefore be pointed at the opposite direction to that intended.

Comparing compounds **1** and **6**, it is evident than PA–CAM conjugates bearing a flexible linker are more efficient than those having a rigid linker. Finally, replacement of the dichloroacetyl moiety of CAM by 1,2,4-triazole-3-carboxylate does not render CAM more efficient (compare CAM with compound **8**). Increasing the distance between 1,2,4-triazole-3-carboxylate and CAM scaffold by a 2-carbon linker further disfavors the formation of CI complex and its subsequent isomerization to C*I complex (compound **9**).

Although the *K*_i_*k*_7_/(*k*_6_ + *k*_7_) value provides a basis for ranking the inhibitory activity of PA–CAM conjugates in the puromycin reaction, they were found of minimal value in predicting the antibacterial potency of the tested compounds. This is likely due to the fact that ribosome targeting by an agent is a multistep process involving internalization of the agent into the bacterial cell. The first barrier that should be overcome is the outer membrane. CAM uses pore-forming porins to gain access to the periplasm in Gram-negative bacteria (47). Normally, conjugation of CAM with PAs should lower the diffusion rate through porins, since the PA portion increases the size of the drug. Moreover, PA portions, due to their polycationic nature, may bind to internal regions of porins and trigger channel closure (48). On the other hand, compounds bearing polycationic components can penetrate the outer-membrane barrier, by using a self promoted uptake pathway that destabilizes the liposaccharide layer (48). The polyamine portion(s) may also endow PA–CAM with the ability to penetrate the second bacterial barrier, plasma membrane, by using the polyamine uptake system, a group of polyamine carriers which pertain to the ATP-binding cassette transporter family (49). In *E. coli* cells, among the constituents of the putrescine-specific uptake system, PotF protein is a periplasmic component that preferentially binds putrescine and is strictly dependent on the integrity of the diaminobutyl portion of the ligand (50). This fact can explain why this uptake system has little contribution to the internalization of compound **1** into the cells. Another periplasmic component of the spermidine/spermine-preferential uptake system, PotD protein, successfully recognizes PAs analogues with intact aminopropane portion(s). Benzyl substitution of the *N*8 amino group of spermidine or spermine has been found to largely contribute to their affinity for this transporter (51). These observations prompted us to assume that compounds **4** and **5** receive better recognition by the PotD protein, than non-benzylated PA–CAM conjugates (compounds **2**, **3** and **7**). The relatively higher ratio IC_50_/IC_50(puro)_ calculated for compound **5**, compared to those for compound **4**, may be explained by the fact that only compound **4** possesses an intact aminopropane portion. On the other hand, it could be expected that compound **5**, as being more basic than **4**, would be better sequestered by efflux pumps whose the substrate binding pocket is rich in acidic residues (52).

To formulate a hypothesis explaining the kinetic data, complexes CI and C*I of compounds **4** and **5**, the most potent members among the PA–CAM conjugates, were structurally characterized by footprinting analysis. As shown in Figure 4, and numerically illustrated in Table 2, the footprinting pattern of complex CI for both compounds does not significantly differ from that previously described for CAM (4). Such a pattern is consistent with PA–CAM conjugates occupying, via their CAM portion, a site placed within the crevice of the ribosomal A-site, and similarly to CAM, interfering competitively with the binding of puromycin. Due to technical limitations, possible interactions well characterized by crystallography (31,46,53) or mutagenesis studies (15–17), like those with C2452 and A2503, cannot be revealed by footprinting analysis, because the first nucleobase does not react with DMS (4), while the latter one is masked by a post-transcriptional methylation at position 2′ of adenosine (16). Accommodation of compound **4** at its final position (complex C*I) results in strong protections at U2585, A2062 and A2059, as well as in an increase of the reactivity of A2058 against DMS. These changes cannot be attributed to a late binding of a second molecule of compound **4**; according to the kinetic model shown in Scheme 1, binding of the drug to the initial (complex CI) and the final position (complex C*I) is mutually exclusive. On the other hand, only minimal translocation events could be assumed for the CAM scaffold, taking into account that its principal interactions with nucleosides clustered around the A-site (A2451, U2506 and G2505) are preserved. Taken together, it could be hypothesized that, as compound **4** seeks out its final position, changes in the conformation of the PTase center allow the interaction of compound **4** with a flexible nucleoside, A2062, placed midway between the PTase center and the entrance to the exit tunnel. This interaction could then trigger allosteric effects on the exit tunnel, transmitted through a signal exchange network comprising nucleosides m^2^A2503, A2059 and A2058. As anticipated by other investigators (54–56), even small changes in the conformation of the hydrophobic crevice A2057-A2059 could affect functions of the PTase center, justifying its pivotal role in serving as a drug sensor. The protection seen at U2585 can be attributed to interactions of compound **4** with the ribosome, via the benzylated terminal aminogroup of spermidine; footprinting analysis of compound **3**, the structurally counterpart of **4** lacking such a substitution, fails to detect a similar protection (Supplementary Figure S6). Corroborative evidence comes from molecular modeling of compound **4** in complex with the 50S ribosomal subunit of *E. coli*. As shown in Figure 5A, the CAM moiety of compound **4** keeps unbroken most of its interactions with adjacent nucleosides, previously revealed by crystallography (31). Moreover, the carbonyl group of the linker is hydrogen bonded to the exocyclic 6-amino group of A2062. Additional stability is gained from *π*-stacking interactions of the two benzyl rings at the polyamine end with the aromatic rings of U2585 and U2586. Protection effects on U2586 were not expected, as this nucleoside does not react with CMCT (57). U2585 is a universally conserved nucleoside, which along with A2602 control the rotary motion of the 3′-acceptor end of a bound aminoacyl-tRNA, as it spirally rotates around a local 2-fold rotation axis in seeking out its functional orientation toward the P-site bound peptidyl-tRNA (58). Therefore, loss of the required flexibility of U2585 may be detrimental for peptide-bond formation. The interaction with U2585 and U2586, a late event during the binding process, renders the binding of compound **4** to the ribosome more stable than those of CAM and can explain its superior activity against resistant bacterial strains (Table 3). The interaction energy (electrostatic + VdW) between compound **4** and nucleosides of 23S rRNA placed within a distance of 6.0 Å was computationally calculated over the last 1000 frames of simulation to be −204.3 ± 12.0 kcal/mol.

The footprinting pattern of C*I for compound **5** generally resembles that of compound **4.** However, two distinct traits make the difference: first, the changes seen in the reactivity of A2058, A2059, and A2062 against DMS are lost, and second, the protection effect on U2585 becomes not statistically significant. Consistently, molecular modeling shows that interactions with A2062 and U2585 are impossible; however, the stacking of the polyamine benzylated tail with U2586 is preserved (Figure 5B). The inability of compound **5** to interact with A2062 explains why conformational rearrangements of A2058 and A2059 are also absent. Since contacts between compound **5** and A2062 and U2585 residues of 23S rRNA are lost, the interaction energy between compound **5** and its 6.0 Å neighborhood is increased to -123.6±29.6 kcal/mol. To compare binding data of compounds **4** and **5** with those of CAM, MD simulations were done for CAM binding and the data along with a recent crystallographic model of CAM complexed with the *E. coli* ribosome (31) are shown in Figure 5C. CAM fairly retains its crystal structure and position, with a Root Mean Square Deviation (RMSD) equal to 0.880 Å. Its interaction energy was calculated to be -88.8 ± 5.4 kcal/mol.

Compound **4**, the most potent PA–CAM conjugate, had little or no effect on the viability of human monocytes and lymphocytes during the 120 h culture period, whereas it displayed a moderate and transient toxicity to neutrophils. As previously suggested for the CAM toxicity (59), these effects may be attributed to an early induction of ROS generation by compound **4**, which are then alleviated by the activation of antioxidant defense mechanisms. With regards to the toxicity of compound **4** on leukemic cell lines, our results indicated that compound **4** was more effective than CAM in inhibiting Jurkat and HS-Sultan cell proliferation. Compound **4**, at 60 μM, induced necrosis in HS-Sultan and Jurkat cells and apoptosis in HS-Sultan cells, in contrast to CAM that failed to induce necrosis/apoptosis in Jurkat cells and apoptosis in HS-Sultan cells.

Contrary to CAM, compound **4** was also found to selectively kill ZL34 cells, with respect to immortalized human mesothelian cells. Penetration of ZL34 cells by **4** was competitively inhibited by exogenously added spermidine, a finding suggesting that import of **4** into cells is facilitated by means of the PAT system. Although particular attention was paid to adopt most of the conclusions drawn by related studies (27,60–64), we chose to use native polyamine motifs, instead of the homospermidine architecture that has been identified as an excellent vector system in mammalian cells (27,62), because our PA–CAM conjugates had in parallel to enter prokaryotic cells. Once imported in mammalian cells, a putative target of PA–CAM conjugates would be the mitochondrion, due to its highly negative membrane potential. As previously detected, destabilization of mitochondrial membranes and/or inhibition of mitochondrial protein synthesis may promote release of organelle's components and/or ROS generation, leading to apoptosis (26) or autophagy (65,66). In fact, CAM itself is a well-documented example of a drug that inhibits both bacterial and mitochondrial translation (20). Alternatively, or in addition, PA–CAM conjugates may induce the spermidine/spermine *N*^1^-acetyltransferase activity, thus leading to depletion of native polyamine pools and cell death (67). Future studies will resolve whether these new agents act via induction of mitochondrial translation stress or other mechanisms.

In conclusion, our study demonstrates that conjugation of CAM with PAs not only adds a novel application in the series of Trojan horse approaches, but also enriches CAM with additional reactive groups that improve its antibacterial and anticancer properties. Time-resolved footprinting analysis and molecular dynamics rationalize the kinetic data, help in definition of dynamical features governing the recognition and accommodation of PA–CAM conjugates by the ribosome, and provide a starting point for optimization of their structures.

## SUPPLEMENTARY DATA

Supplementary Data are available at NAR Online.

SUPPLEMENTARY DATA
