# Supplementary material for: Conjugation with polyamines enhances the antibacterial and anticancer activity of chloramphenicol
Source: Nucleic Acids Res. 2014 Jun 26;42(13):8621–34. doi: 10.1093/nar/gku539 (PMC4117768; doi:10.1093/nar/gku539)
Supplement: SUPPLEMENTARY DATA [file supp_42_13_8621__index.html]

Conjugation with polyamines enhances the antibacterial and anticancer activity of chloramphenicol — Conjugation with polyamines enhances the antibacterial and anticancer activity of chloramphenicol — Conjugation with polyamines enhances the antibacterial and anticancer activity of chloramphenicol — SUPPLEMENTARY DATA 

# Conjugation with polyamines enhances the antibacterial and anticancer activity of chloramphenicol

## SUPPLEMENTARY DATA

**Files in this Data Supplement:**

- SUPPLEMENTARY DATA
