# Supplementary material for: Conjugation with polyamines enhances the antibacterial and anticancer activity of chloramphenicol
Source: Nucleic Acids Res. 2014 Jun 26;42(13):8621–34. doi: 10.1093/nar/gku539 (PMC4117768; doi:10.1093/nar/gku539)
Supplement: SUPPLEMENTARY DATA [file supp_gku539_nar-01360-f-2014-File008.docx]

**Figure S1.** Toxicity assays in human peripheral blood cells. Peripheral blood was collected in EDTA-coated tubes from 5 healthy volunteers (age range: 25-30 years). Cell concentration was adjusted to 1.8×10^9^ cells/l using RPMI-1640 medium (GIBCO BRL) containing 1% penicillin/streptomycin. CAM or compound **4** was added at final concentrations equal to 30 or 60 μM and cells were cultured in triplicate under a humidified 5% CO_2_ atmosphere for 5 days, at 37°C. Control cultures were carried out in the absence of CAM or compound **4**. Cultures were counted daily in a CELL-DYN 3700 Hematology Analyzer and values are expressed as a percentage of cells measured in control cultures.


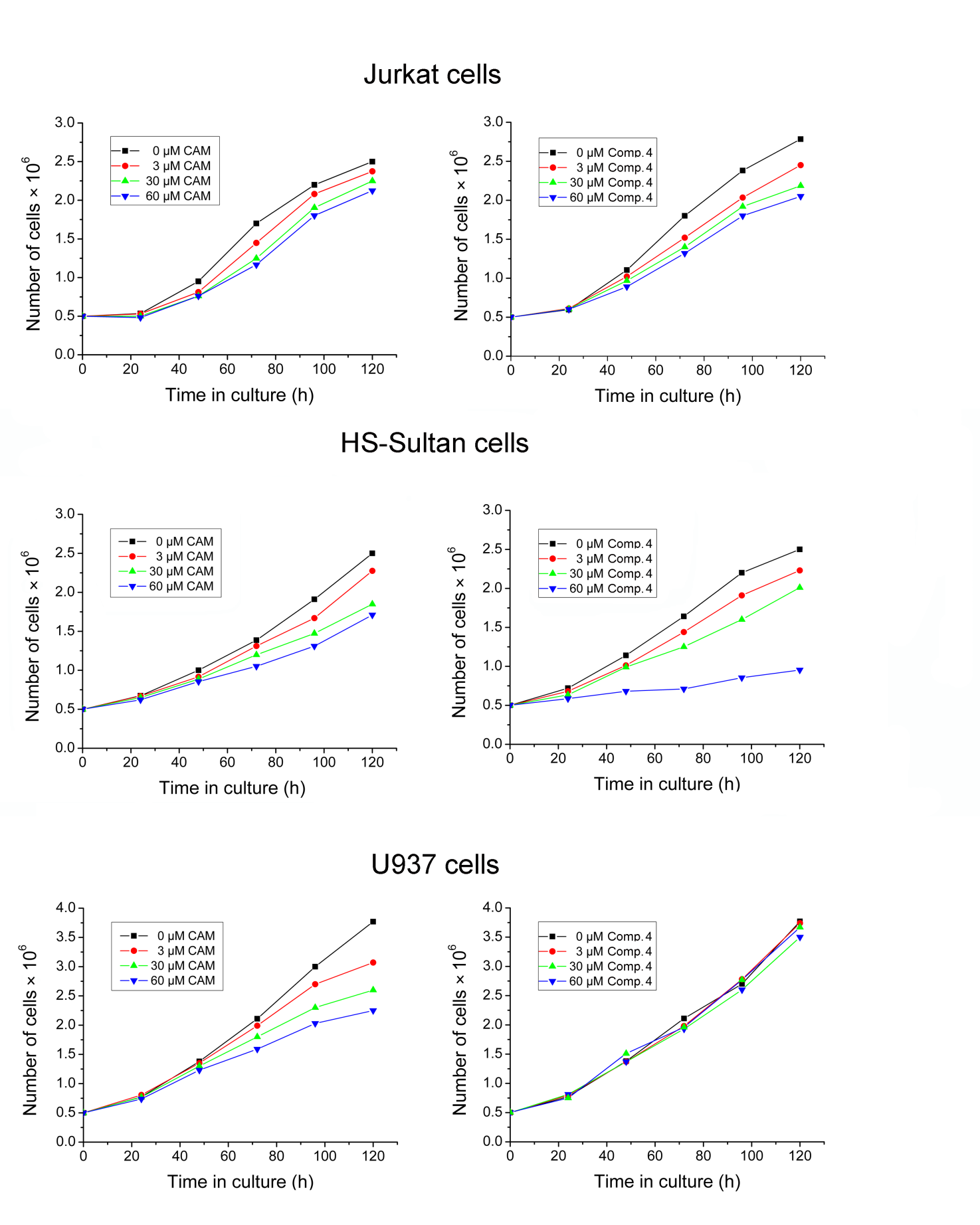


**Figure S2.** Toxicity assays in leukemic cell lines. Human leukemic cell lines, HS-Sultan, Jurkat, and U937 were adjusted to 1×10^9^ cells/l in RPMI-1640 medium containing 1% Penicillin/Streptomycin and 10% fetal bovine serum and grown in triplicate in the presence or absence of CAM or compound **4** at the indicated concentrations for 5 days at 37°C, under a humidified 5% CO_2_ atmosphere. Aliquots were collected daily and counted in a CELL-DYN 3700 Hematology Analyzer.

^
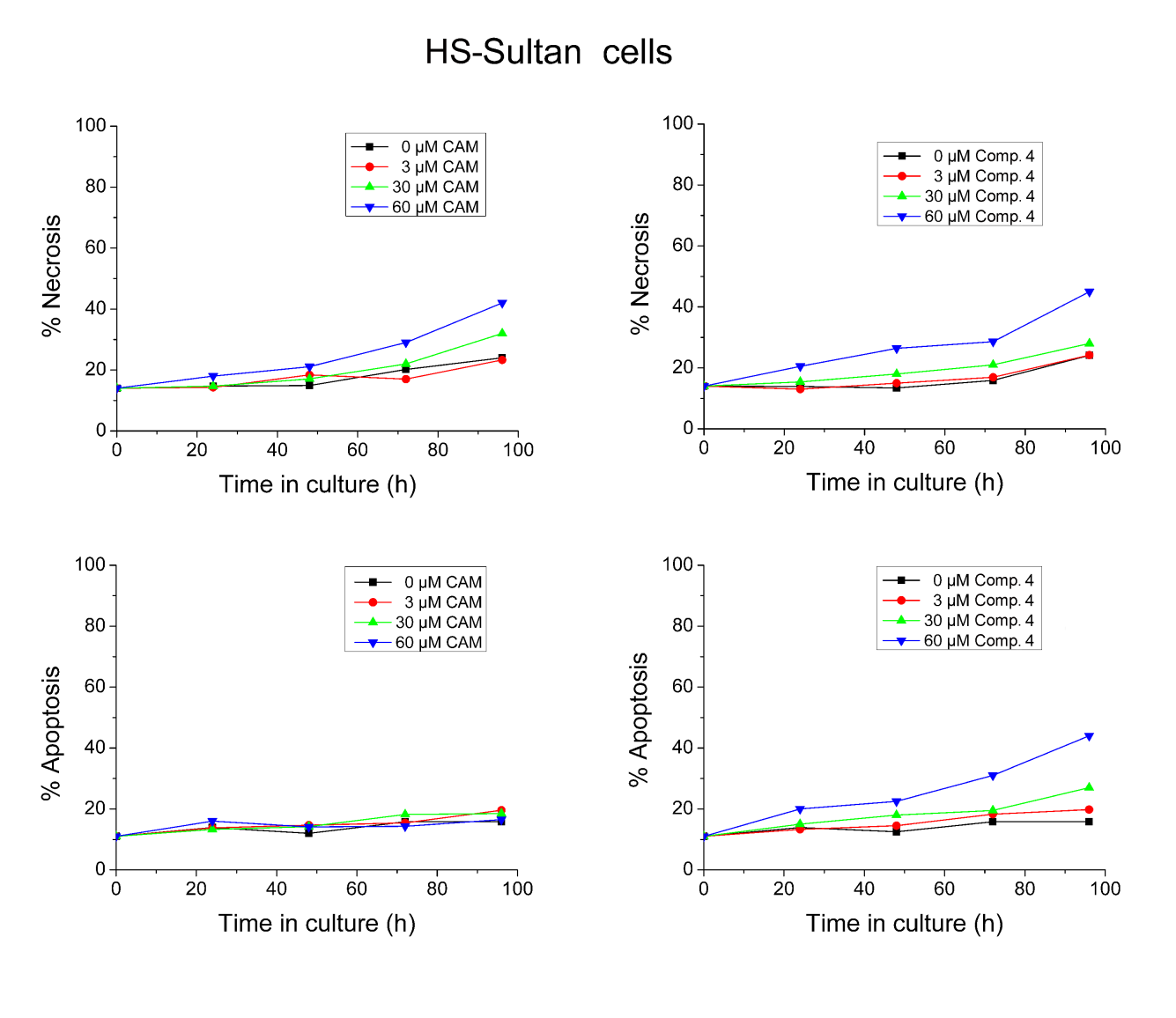
^

**Figure S3.** Toxicity assays in HS-Sultan cells. HS-Sultan cells were adjusted to 1×10^9^ cells/l in RPMI-1640 medium containing 1% Penicillin/Streptomycin and 10% fetal bovine serum and grown in triplicate in the presence or absence of CAM or compound **4** at the indicated concentrations for 4 days at 37°C, under a humidified 5% CO_2_ atmosphere. For cell necrosis and apoptosis assays, samples (10^6^ cells) were collected daily and determined using the Annexin V-PE Apoptosis Detection Kit I for flow cytometry. Non-viable and necrotic cells were expressed as a percentage of total cells.

**
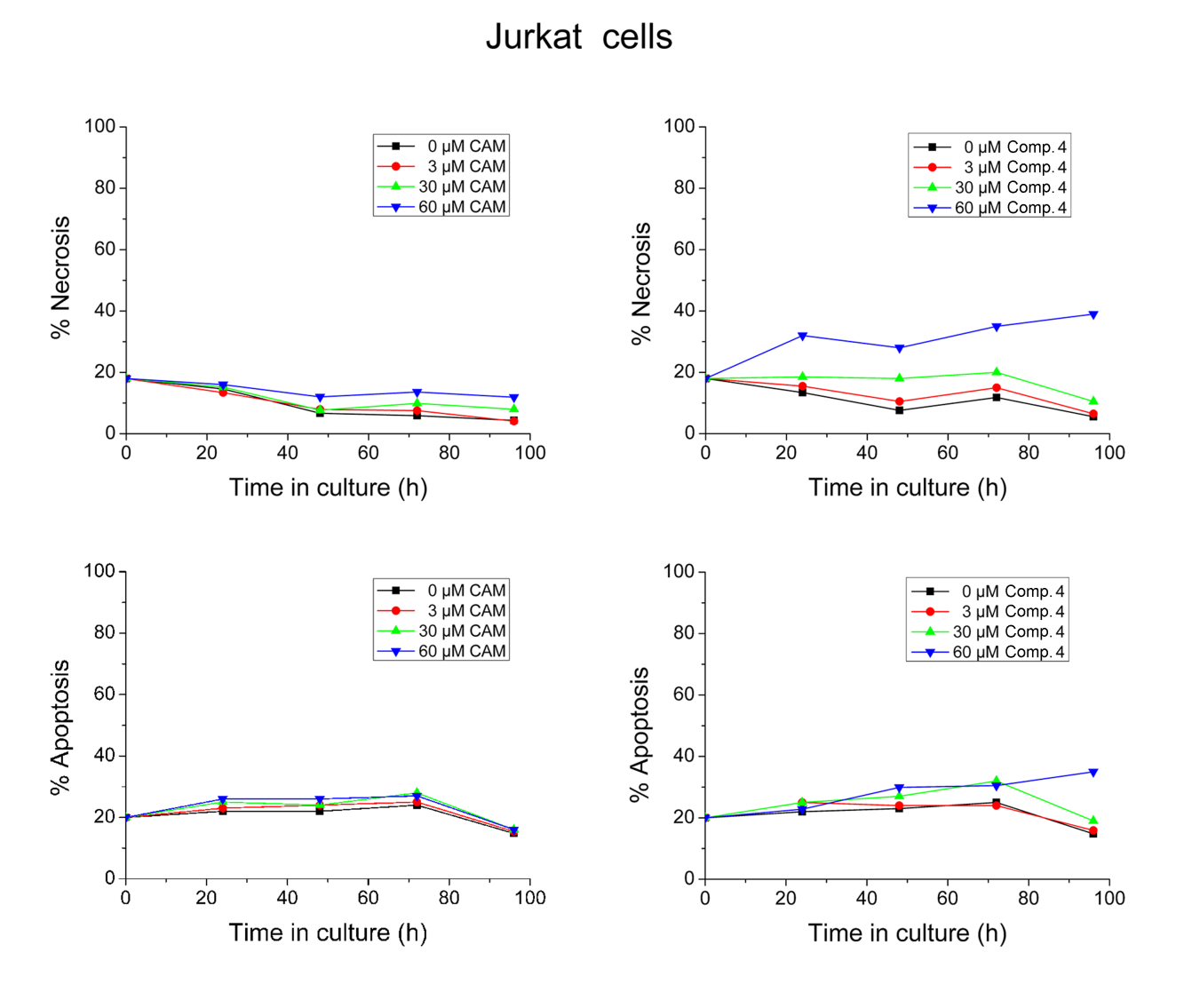
**

**Figure S4.** Toxicity assays in Jurkat cells. Jurkat cells were adjusted to 1×10^9^ cells/l in RPMI-1640 medium containing 1% Penicillin/Streptomycin and 10% fetal bovine serum and grown in triplicate in the presence or absence of CAM or compound **4** at the indicated concentrations for 4 days at 37°C, under a humidified 5% CO_2_ atmosphere. Cell necrosis and apoptosis assays were performed as described in Figure S3. Necrotic and apoptotic cells were expressed as a percentage of total cells.





**Figure S5.** Toxicity assays in ZL34 and Met5A cell lines. ZL34 or Met5A cells were plated in sterile 96-well microtiter plates at 5×10^4^ cells/ml and grown in DMEM, supplemented with 5% fetal bovine serum. Cultures were maintained in a humidified atmosphere with 5% CO_2_, at 37°C. Solutions at the appropriate concentration of each compound were added, and then cells were grown for 24 h, 72 h and 96 h. After treatment, CAM or compound **4** was removed by washing the cells twice with PBS. The cells were then trypsinized, mixed with 1 ml DMEM and collected by centrifugation at 3,000×*g* for 5 min. Cell viabilities were determined by the trypan blue exclusion assay, using a TC10 automated cell counter (BIO-RAD). Viable cells were expressed as a percentage of total cells. SPD, spermidine.

**

**

**Figure S6.** Protection against CMCT in nucleotide U2585 of the central loop of domain V of 23S rRNA, caused by binding of compound **3** to *E. coli* ribosomes. Ribosomes were incubated in the presence or absence of compound **3** at 25°C for 2 s or 3 min. The resulting complexes were then probed with CMCT. U, A, G and C, dideoxy sequencing lanes; lane 1, unmodified ribosomes; lane 2, ribosomes probed in the absence of compound **3**; lane 3, ribosomes pre-incubated with compound **3** for 2 s and then probed; lane 4, ribosomes pre-incubated with compound **3** for 3 min and then probed. Results obtained with CAM are presented in lanes 5-7, for the sake of comparison. Numbering of nucleosides for the sequencing lanes is indicated at the left. Stop of reverse transcriptase at the modified nucleotide U2585 is shown by an arrow at the right, while a reference band whose intensity is not affected by compound **3** or CAM binding is indicated by an asterisk. The relative intensity of the reference band, that corresponds to a natural stop in reverse transcription at nucleotide C2612 (57), was used to correct the variability between lanes (horizontal normalization).

**Table S1.** Determination of the ratio IC_50_/IC_50(puro)_ in wild-type *E. coli* for CAM and PA-CAM conjugates, indicating how much the *in vitro* and *in vivo* inhibitory activities differ ^a,b^

| Compound | IC_50_/IC_50(puro)_ |
| --- | --- |
| **CAM** | 1.17 ± 0.13 |
| **1** | >37.0 |
| **2** | >22.2 |
| **3** | >20.8 |
| **4** | 5.60 ± 0.76 |
| **5** | 9.86 ± 1.29 |
| **7** | >3.3 |
| **8** | >50 |
| **9** | >8.3 |

^a^Data represent the mean±SE values obtained from three independently performed experiments, with two replicates per experiment.

^b^The IC_50_ values were taken from Table 3. The term IC_50(puro)_ is defined as the compound concentration causing 50% inhibition in peptide-bond formation at the presence of 2 mM puromycin, and its value was calculated through the relationship,

$${IC}_{50\left( puro \right)}=K_{i}^{*}(1+ \frac{\left[ S \right]}{K_{S}} )$$

High value of the ratio IC_50_/IC_50(puro)_ means that the *in vivo* inhibitory activity of the corresponding compound is much less than that the *in vitro* activity predicts.
